# The value of non-invasive prenatal testing: preferences of Canadian pregnant women, their partners, and health professionals regarding NIPT use and access

**DOI:** 10.1186/s12884-018-2153-y

**Published:** 2019-01-10

**Authors:** Stanislav Birko, Vardit Ravitsky, Charles Dupras, Jessica Le Clerc-Blain, Marie-Eve Lemoine, Aliya O. Affdal, Hazar Haidar, Anne-Marie Laberge

**Affiliations:** 1 University of Montreal School of Public Health, Montreal, Canada; 2 Centre of Genomics and Policy, McGill University, Montreal, Canada; 3 CHU Sainte-Justine Research Center, Montreal, Canada; 4 University of Montreal Ethics Research Centre, Montreal, Canada

**Keywords:** NIPT, Non-invasive prenatal testing, Prenatal screening, Public policy, Insurance coverage, Reproductive decision-making, Testing pathway, Equal access

## Abstract

**Background:**

Canadian policies regarding the implementation and public coverage of non-invasive prenatal testing (NIPT) are heterogeneous and shifting, with NIPT being publicly covered for high-risk pregnancies in some provinces, but not others. Such a diverse and evolving policy landscape provides fertile ground for examining the preferences of pregnant women, their partners, and health professionals regarding the implementation and coverage of NIPT by the public healthcare system, as well as the factors influencing their preferences, which is what the present study does.

**Methods:**

In this paper, we report the results of three-large scale Canadian surveys, in which 882 pregnant women, 395 partners of pregnant women, and 184 healthcare professionals participated.

**Results:**

The paper focuses on preferences regarding how and when NIPT should be used, as well as the factors influencing these preferences, and how coverage for NIPT should be provided. These are correlated with respondents’ levels of knowledge about Down syndrome and testing technologies and with their stated intended use of NIPT results.

**Conclusion:**

Salient is the marked difference between the preferences of prospective parents and those of healthcare professionals, which has potential implications for Canadian policy regarding NIPT implementation and insurance coverage.

**Electronic supplementary material:**

The online version of this article (10.1186/s12884-018-2153-y) contains supplementary material, which is available to authorized users.

## Background

Non-Invasive Prenatal Testing (NIPT)^1^ is an emerging technology aiming to detect fetal aneuploidies such as trisomies 21, 18 and 13 through the analysis of cell-free DNA (cfDNA) originating from the placenta and present in maternal blood. Performed as early as 9 weeks of pregnancy, NIPT holds no risk of miscarriage and offers clinical benefits over existing prenatal screening tests, such as maternal serum screening (MSS), by detecting the presence of trisomy 21 (Down syndrome, DS) with high sensitivity (99.9%) and specificity (98%) [1].

NIPT was first offered in 2011 and in the early days it was thought to have the potential to rapidly become a diagnostic test that would replace invasive testing methods posing risk to the fetus. Over time, professional societies recommended it as a second-tier screening test for women already identified as having a high-risk of trisomy based on traditional screening tests [2]. More recently, some have concluded, based on emerging data, that the technology is ready for implementation as a first-tier screening test for all pregnant women [3, 4]. The current mainstream use of NIPT in Canada remains as a second-tier screening test offered to women who have undergone first-tier traditional screening and have been identified as having a high risk of trisomy.

In 2011, the Society of Obstetricians and Gynecologists of Canada (SOGC) recommended that any prenatal screening test offered to Canadian women should have, at minimum, a detection rate of 75.0% with no more than a 3.0% false-positive rate in the first trimester and a detection rate of 75.0% with no more than a 5.0% false-positive rate in the second trimester [5]. Each province/territory devised their own screening program, so the specific screening test used varies across Canada.

In 2013, the Genetics Committee of the SOGC recommended that NIPT be offered to pregnant women who have been identified as being at increased risk of fetal aneuploidies, through the screening available in their province/territory, i.e. as a second-tier screening test [6]. In 2014, the International Society for Prenatal Diagnosis (ISPD) considered the offer of NIPT as a first-tier screening test for all pregnant women to be an “appropriate” option [7]. However, concerns regarding sensitivity, specificity and positive predictive value remain [8]. Moreover, the cost of NIPT in 2018 in Canada - C$300 to C$500 – can create a barrier to access for many prospective parents, which in turn, raises issues of equity of access and justice. Such issues may be mitigated by ensuring NIPT is publicly funded. Currently, in Canada, only the provinces of Ontario and British Columbia and the territory of Yukon [9] have decided to reimburse the test under certain conditions, i.e., only for pregnant women at high risk of fetal aneuploidies, and at the time of the study, only Ontario was reimbursing the test [10]. In the other provinces, patients need to pay for the test out of pocket or through private insurance.

The cost-effectiveness analysis of NIPT and its introduction into the public healthcare system have been subject to studies in several countries such as Italy [11], Australia [12], Sweden [13], United States [14] and Canada [15]. However, little is known about the preferences of different Canadian publics regarding NIPT implementation and coverage. Results presented here come from the first large-scale study of Canadian pregnant women, their partners and health professionals regarding their perceptions of and attitudes towards NIPT. This study was part of a pan-Canadian research project titled “Personalized Genomics for prenatal Aneuploidy Screening Using maternal blood” (or PEGASUS), aiming to “validate the performance and utility of [NIPT] for screening for major fetal chromosome imbalances”. This paper focuses specifically on the preferences of pregnant women, their partners, and health professionals regarding the implementation and coverage of NIPT by the public healthcare system.

## Methods

The study consisted of three surveys aimed at three populations: pregnant women, their partners, and health professionals. It ran during a 16-month period, from March 2015 to July 2016. Recruitment occurred at 5 Canadian sites in Alberta, BC, Ontario and Québec, where the PEGASUS study ran, as well as one additional site in Newfoundland & Labrador.

All pregnant women and their partners attending a routine appointment regarding their current pregnancy at one of these sites during that period were eligible to participate. Aside from being currently pregnant, there were no other explicit inclusion criteria. No incentive for participating was provided (aside from the general incentive of furthering knowledge inherent in any study). Respondents were provided with a paper copy of the questionnaire, which included a URL for an online version. Health professionals were recruited at conferences, at the 6 sites participating in the study, and via mailing lists of 10 Canadian professional societies.

### Questionnaire development

Questionnaires were developed based on a literature review and questionnaires used in previous studies [16–22]. The questionnaires for pregnant women and partners were reviewed by the PEGASUS team and by Lyn Chitty for face and content validity, and then piloted on 8 women of reproductive age, followed by cognitive debriefing. Based on feedback, the questionnaires were modified for clarity and length before being finalized. Questionnaires for health professionals were adapted from these questionnaires and piloted on 4 health professionals and 1 clinical research coordinator from a university medical center.

**Fig. 1:**
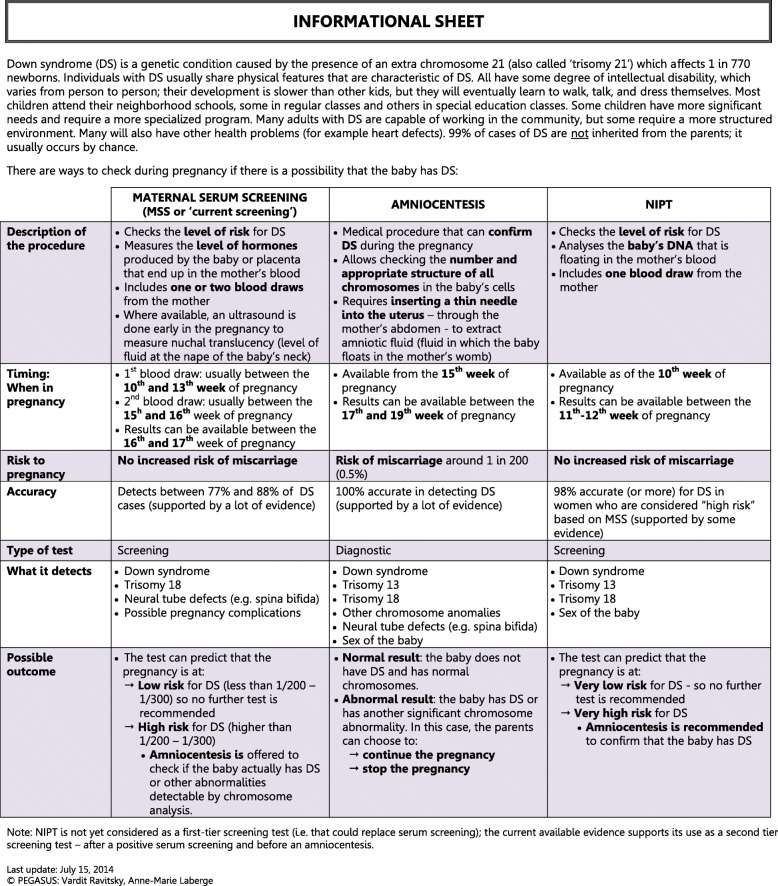
Informational Sheet. Information regarding different prenatal testing technologies provided to all study participants together with the questionnaire

The pregnant women’s questionnaire (41 questions, Additional files 1) and partners’ questionnaire (43 questions, Additional files 2) explored the same themes: knowledge about DS and NIPT, informed consent, uses of NIPT, decision-making and the involvement of others, social impact of NIPT, and future uses of NIPT. The health professionals’ questionnaire was shorter (28 questions, Additional files 3) but addressed similar themes. All surveys collected relevant socio-demographic characteristics. Question formats included Likert scales, ‘true or false’ statements, multiple choice, and ranking. The questionnaire was distributed along with an information sheet explaining the differences between MSS, amniocentesis and NIPT. The information sheet gave brief descriptions of the procedures, timing of tests, risk for pregnancy, accuracy, nature of test (screening vs diagnostic), potential results, and potential outcomes (see Fig. 1. Info Sheet).

### Data analysis

Data were stored and analyzed using IBM SPSS 24 [23]. To test the level of influence different factors have on participants’ attitudes towards NIPT use and coverage, their responses were analyzed based on socio-demographic characteristics, knowledge of DS and available testing options, and their stated intended use of test results. Statistical analysis was done using Pearson Chi-Square tests, Kruskal-Wallis and Mann-Whitney U tests, Spearman’s rho and Kendall’s tau correlations where applicable. To account for multiple testing, *p* < 0.001 (two-sided) was established a priori as the threshold of statistical significance reported.

### Ethical considerations

Ethics approval for the study (including the consent procedure) was obtained from *Comité d’éthique de la recherché* of the *CHU Sainte-Justine* associated with the University of Montreal (#3781) as well as locally from each of the following: the *Comité d’éthique de la recherché du CRCHU de Québec*, the *Ottawa Health Science Network Research Ethics Board*, the *UBC Children’s and Women’s Research Ethics Board*, the University of Calgary’s *Conjoint Health Research Ethics Board*, and the *Newfoundland and Labrador Health Research Ethics Authority*. The latter REB required a psychology professional to be available, citing concerns about the study raising anxiety. By completing and submitting the completed questionnaire, respondents confirmed their consent to participate, as the questionnaire’s cover page made explicit.

## Study results

### Socio demographic data

A total of 882 pregnant women, 395 partners of pregnant women, and 184 health professionals participated in the study. Assuming 380,000 annual births in Canada, this yields a maximum margin of error of 3.29% for the pregnant women sample, 4.93% for the partner sample (assuming all pregnancies involved a partner). In 4 of the 6 sites for pregnant women and their partners, the number of pregnant women approached was kept track of – 1603. Of these 1603, 755 completed the questionnaire, yielding a response rate of 47.1%. All pregnant women who were approached were also given a survey for their partner. Of the 1603 couples approached, 366 partners completed the survey (22.8% or more considering that not all pregnant women with a partner necessarily offered the survey to their partner). Demographic characteristics of participants are summarized in Tables 1 and 2.

**Table 1:** Pregnant Women and Partners’ Characteristics

Characteristic	% of Pregnant Women *n* = 882	% of Partners *n* = 395
Age Mean (SD)	32.3 (4.8)	33.5 (5.7)
Gender (of partner) Other than male	N/A	2.8
Province of Residence		
BC	28.5	6.1
AB	13.2	7.1
MB	0.2	0
ON	13.4	19.7
QC	43.9	66.8
Atlantic provinces	0.2	0
Territories (Nunavut/NWT/Yukon)	0.2	0
Country of birth		
Canada	80.3	86.8
Language Mostly Spoken at Home		
English	54.2	33.9
French	40.2	65.1
Other	4.2	3.0
Race/ethnicity		
Caucasian/white	81.4	86.1
North American Aboriginal (First Nation, Inuit, Metis)	1.6	2.3
Other	17.0	11.6
Religion/culture		
Christian	55.8	60.0
Muslim	2.2	2.0
Buddhist	1.8	0.8
Jewish	1.4	0
Hindu	0.8	0
Sikh	0.6	0.5
None/agnostic/atheist/spiritual	39.9	38.2
Importance accorded to religion/spirituality (1–5)		
Mean (SD)	2.26 (1.25)	1.92 (1.19)
Highest completed education level		
Elementary School	0.2	1.3
High School	7.0	14.6
Trade School	5.3	12.8
CEGEP/College	19.8	18.9
University	66.9	52.4
Are you a Healthcare Professional: Yes	26.5	7.8
Relationship Status		
Married	57.9	40.0
Common-law	37.9	53.9
Single	2.6	2.5
Divorced/separated	0.3	0.5
Other	0.5	1.0
Already has a child	45.8	39.0
With Down Syndrome	0.6	0.3
With physical or intellectual disability	1.0	0.3
Does anyone close to you have a child with Down Syndrome	7.7	9.0
Current pregnancy is		
Low-risk for DS	64.1	60.1
High-risk for DS	11.2	11.0
Unsure	23.5	28.9
Current pregnancy was conceived		
Naturally	89.6	91.3
Using IVF	5.4	4.4
Using ART other than IVF	2.7	4.4
Has had prenatal screening in a previous pregnancy	32.5	25.7
Has had prenatal diagnosis (chorionic villus sampling or amniocentesis) in a previous pregnancy	6.7	8.2
Who provided you info re current screening, NIPT or amnio		
Family physician	41.2	28.6
Ob/gyn	31.4	25.3
Nurse	15.9	20.2
Midwife	12.4	4.3
Genetic counsellor	7.9	8.4
Medical geneticist	6.9	9.1
Other (mostly the pregnant partner)	N/A	33.2

**Table 2:** Healthcare Professionals’ Characteristics

Characteristic	% of Health Professionals *n* = 184
Age	
Mean (SD)	41.8 (10.3)
Gender	
Female	78.3
Male	19.0
Main field of practice	
Genetic counselor	29.3
Obstetrician Gynecologist	28.8
Clinical geneticist	9.8
Nurse	6.5
Midwife	5.4
General Practitioner	3.8
Other	16.4
Years of practice	
Mean (SD)	12.4 (9.5)
Province of practice	
BC	15.8
AB	13.0
MB	0.5
ON	35.3
QC	28.3
Atlantic Provinces	3.8
Territories (Nunavut/NWT/Yukon)	0.5
Practice environment	
Public hospital	50.0
Research hospital	20.7
Private practice	15.2
Public health organization	5.4
Other	8.7
Years of experience in prenatal setting	
Mean (SD)	10.6 (9.3)
Approx. # of prenatal patients seen in prenatal setting per week	
Mean (SD)	28 (76)
Approx. % of patients at ‘high risk’ for Down syndrome	
Mean (SD)	26.7 (28.3)
Experience in prenatal diagnosis for Down syndrome	87.5
Currently offering NIPT	73.6
Down syndrome screening currently offering	
Integrated prenatal screening (IPS)	62.7
NIPT	55.1
First trimester screening	38.9
Quad screening	30.8
Serum IPS	30.8
Triple screening	12.4

### Knowledge of Down syndrome and testing technologies by pregnant women and their partners

Pregnant women and partners were asked 6 true or false questions regarding DS and 7 regarding different prenatal testing options available to pregnant women who want to know more about the risk of DS for their pregnancy (see Additional files 1, 2 and 3 for the questions used in the three questionnaires). For women, the average score was 5.2/6 for the questions about DS, ranging from 1/6 (0.2%) to 6/6 (47.0%), and 5.4/7 for the questions about prenatal testing, ranging from 1/7 (0.7%) to 7/7 (20.7%). For partners, the average score for the questions about DS was 4.9/6, and 4.9/7 for the questions about prenatal testing.

### Stated intended use of NIPT results by pregnant women and their partners

Pregnant women and partners were asked how they would use information about their fetus having DS. 52.9% of the pregnant women and 56.7% of partners stated that they “would consider terminating the pregnancy if the baby was diagnosed”. 27.1% of the pregnant women and 21.8% of partners wanted “to know in advance to prepare for the birth of the baby”. 14.3% of the women and 15.2% of partners were “unsure” how they would use such information. 2.3% of the women and 2.4% of partners “did not want to know”.

### Attitudes of pregnant women and their partners regarding use of NIPT

In order to gauge preferences about how and when NIPT should be used, as well as the reasons behind these preferences, respondents were asked to make choices based on two vignettes. In the first vignette, a 40-year-old 10-week pregnant woman meets her doctor for her first prenatal visit. After the doctor explains current screening as well as NIPT, the respondent was asked to choose between current screening, NIPT or no testing, as if they were in this woman’s place. The majority of pregnant women (78.3%) chose NIPT as first-tier screening in this scenario while 20.0% opted for current screening, and 1.7% preferred not to screen at all. Partners’ results were comparable, with 80.5% choosing NIPT, 16.1% opting for current screening and 3.4% preferring not to screen.

The extent to which 4 different test characteristics influenced the choice of test was assessed using a 5-point Likert scale, ranging from ‘did not influence’ (1) to ‘strongly influenced’ (5). The average weight of each characteristic for pregnant women who chose NIPT and current screening is given in Table 3.

**Table 3:** Extent to which test characteristics affect decision vs preferred screening and diagnostic technology (numbers are averages of scores on 1–5 Likert scales, with 1 – disagreement, 5 – agreement)

Test characteristics	Women choosing NIPT	Women choosing current screening	% of all women for whom this test characteristic ‘strongly influenced’ their decision	*p*-value comparing the 2 groups
NIPT is much more accurate than current screening in assessing the risk of DS	4.64	3.11	64.5%	< 0.001
Results of NIPT can be available earlier in the pregnancy than the result of current screening	4.57	2.94	62.9%	< 0.001
Current screening estimates the risk that the baby has neural tube defects and NIPT doesn’t	2.55	4.11	15.9%	< 0.001
Current screening can indicate the possibility of pregnancy and labor complications and NIPT cannot	2.40	3.98	13.4%	< 0.001
		Women choosing amniocentesis		
With NIPT there is no increased risk of miscarriage	4.78	2.93	66.5%	< 0.001
NIPT is more convenient than amniocentesis (only requires a blood draw)	4.24	2.61	46.9%	< 0.001
NIPT tests for the common chromosome disorders (like Down Syndrome), which is all I need to know	3.89	2.43	28.5%	< 0.001
Amniocentesis is more accurate than NIPT	2.46	4.61	24.2%	< 0.001
Amniocentesis gives more information about possible chromosome anomalies than NIPT	2.53	4.57	21.9%	< 0.001

As demonstrated in Table 3, women who prioritized accuracy of the test or its timing (i.e., at what time during the pregnancy results could be obtained) were more likely to select NIPT as their preferred first-tier screen, whereas women who prioritized information regarding neural tube defects or pregnancy and labor complications were more likely to opt for current screening. Overall, test accuracy and timeliness ‘strongly influenced’ the decision of more pregnant women than additional information regarding the pregnancy.

The second vignette described a 40-year-old woman who is 16-weeks pregnant and meets her doctor after current screening estimated her pregnancy to be at high risk, i.e., ‘more than 1 in 300 chance that her baby has DS’. A genetic specialist explains her options to be amniocentesis, NIPT, or not undergoing further testing. In this scenario, 72.0% of pregnant women selected NIPT as the next step, 25.6% selected amniocentesis, and 2.4% preferred not to do any further testing. Partners chose NIPT slightly less frequently than pregnant women, with 67.2% preferring NIPT, 27.9% - amniocentesis, and 4.9% preferring not to test.

The influence of five test characteristics on the choice of test was assessed. The results are presented in Table 3. Women who preferred to minimize the risk of miscarriage, who preferred a more convenient test and who were only interested in common chromosome disorders such as DS, chose NIPT as a diagnostic test, even though it was specified that it is less accurate than amniocentesis. Women who prioritized accuracy and greater information regarding chromosome anomalies preferred to undergo amniocentesis rather than NIPT. The only factor ‘strongly influencing’ the majority of pregnant women in their decision was risk of miscarriage. Even of the women who stated that they would consider terminating the pregnancy if the fetus were diagnosed with DS, 61.3% were ‘strongly influenced’ in their decision by the risk of miscarriage.

Women’s preferences regarding use of NIPT (i.e., their choice of test in the two scenarios presented) are associated with who informed them about available testing options. Namely, being informed by a genetic counsellor (*N* = 70 or 7.9% of all respondents) correlated with an equal likelihood of choosing amniocentesis or NIPT, whereas not being informed by a genetic counsellor correlated with being more likely to prefer NIPT (more than 3 times as likely as preferring amniocentesis).

### Attitudes of health professionals regarding use of NIPT

Health professionals were asked what they thought was currently the most appropriate approach to using NIPT among the following choices: 1) ‘Current screening, followed by NIPT as second-tier screening (confirmed with amniocentesis)’; 2) ‘NIPT as first-tier screening (replacing current screening), confirmed with amniocentesis’; 3) ‘NIPT as a diagnostic test (without confirmation by amniocentesis), then availability of pregnancy termination if NIPT result is positive’; and 4) ‘Other’. NIPT as second-tier screening test was the most popular option (50.0% of health professionals), with NIPT as first-tier closely behind (42.4%). Only a small minority considered it appropriate to use NIPT as a diagnostic test (option 3) (2.2%) Finally, 2.2% selected ‘other’ and 3.2% did not answer the question.

### Attitudes of pregnant women, their partners, and health professionals regarding NIPT coverage

Respondents were asked who they thought should have access to NIPT free of charge. The majority of pregnant women (66.9%) said all women should have access to NIPT free of charge. 30.3% believed only women with a high-risk pregnancy should be eligible, 0.2% believed only low-risk pregnancies should benefit from free NIPT, 1.5% believed that nobody should have access free of charge, and 1.1% of pregnant women selected ‘other’ (with at least half of these mentioning the patient’s income as a deciding factor). A slightly smaller proportion of partners (60.5%) believed that all women should have access to NIPT free of charge, while 31.6% of partners believed women with high risk pregnancies should have access to NIPT free of charge. A minority of partners believed NIPT should be free of charge for other groups (2.0% - low risk only, 4.1% - nobody, and 1.8% ‘other’). When asked the same question, 53.3% of health professionals believed only women with a high-risk pregnancy should have access to NIPT free of charge, 39.7% believed all women should have access free of charge, 1.1% - only low-risk pregnancies, 1.1% - nobody, 1.6% - ‘other’, and 3.2% declining to respond. Health professionals’ attitudes were significantly different from those of women (chi-square, *p* < 10^− 10^) and partners (chi-square, *p* < 10^− 9^).

Pregnant women and partners were also asked how much they would be willing to pay for NIPT. They were given 6 ranges of prices ($0, $1–99, $100–499, $500–999, $1000–4999, $5000+). The results are presented in Table 4. Pregnant women’s and partners’ responses are not significantly different (chi-square, *p* = 0.35). No similar question was asked of health professionals.

**Table 4:** Amount Pregnant Women and Partners Would Be Willing to Pay for NIPT

	$0	$1–99	$100–499	$500–999	$1000–4999	More than $5000
All Canadian Pregnant Women	12.3%	36.4%	41.6%	8.4%	1.0%	0.3%
Women from Ontario	22.4%	44.0%	26.7%	5.2%	0.9%	0.9%
All Canadian Partners	15.1%	34.5%	42.5%	6.6%	1.4%	0%
Partners from Ontario	17.1%	42.1%	32.9%	7.9%	0%	0%

Pregnant women and partners were asked how much their decision to use NIPT would be impacted by its being free of charge. 66.4% of pregnant women and 50.3% of partners stated that NIPT being free of charge would have ‘a lot of impact’ (5 on the 1–5 scale) on their decision to use NIPT. Only 5.0% of pregnant women and 11.0% of partners believed this would have no impact whatsoever on their decision (a choice of 1 on the Likert scale). Health professionals were asked to what degree ‘lack of coverage for the test’ constitutes in their opinion a barrier to clinical implementation of NIPT and 66.1% of them rated it at 5 (‘definite barrier’) making it the number 1 barrier (of seven presented), and the only feature of the test (out of the seven presented) to be considered a definite barrier by most respondents. When asked whether NIPT’s coverage would influence their decision to offer NIPT to a specific patient, 50.5% of professionals responded affirmatively.

All three populations were asked whether they would be concerned by increased pressure on women to use NIPT if it were covered as part of routine prenatal care. 62.4% of pregnant women, 51.2% of partners, and 33.7% of healthcare professionals were “not concerned” about such pressure being felt by pregnant women. Conversely, 1.8% of women, 3.3% of partners, and 7.1% of healthcare professionals were “very concerned”.

Of the three questions assessing women’s attitudes towards NIPT coverage, socio-demographic factors only influenced the amount women were willing to pay for NIPT. Older respondents were more likely to be willing to pay more (Kendall’s tau = −.158). Respondents residing in Ontario were more likely to be willing to pay less than residents of other provinces, with the most popular choice (44.0%) among Ontarian women being $1–99 and the most popular choice among the rest of Canadian women being $100–499 (see Table 4). Women living with their partners (whether legally married or common-law) were more likely to be willing to pay more than single women.

Already having a child (or children) with physical or intellectual disabilities affected the amount the respondent reported being willing to pay in a particular fashion. Of the 9 (1.0%) women with children with physical or intellectual disabilities, 3 were willing to pay $0, 3 - $1–99, and 3 - $500–999. While overall, pregnant women’s most popular choice was $100–499, this choice was not made by any woman who had a child with disabilities. Correctly answering questions regarding both DS and testing options correlated with a reported willingness to pay more (Kendall’s tau = .203). Women stating that they intend to use the results of testing to consider terminating the pregnancy if the baby was diagnosed with DS (as well as those unsure how they would use the results) were willing to pay more than those who wanted to know in advance to prepare for the birth of a baby diagnosed with DS.

Being born in Canada or elsewhere; speaking English, French or another language; race/ethnicity; religious/cultural background; importance of religion/spirituality; education level; being a healthcare professional; having children; having a child with DS (*N* = 5, 0.7%); being close to a parent of a child with DS; being of low or high risk for the current pregnancy; using ART to conceive; having had prenatal screening or diagnosis in a previous pregnancy were not significant factors in explaining the differences in attitudes towards NIPT use and coverage.

## Discussion

The present study, to the best of our knowledge, is the first large-scale survey on NIPT that takes into consideration the attitudes of pregnant women, their partners as well as healthcare professionals. It offers evidence of some main stakeholders’ opinions regarding how the test should be used and whether it should be covered by public insurance. While policy decisions regarding the implementation of NIPT (e.g. as second versus first tier screening test) should be based on scientific evidence regarding its performance in various populations (e.g. high versus low risk pregnancies), it is also important to consider the attitudes and preferences of stakeholders regarding the various uses of this technology, as these reflect values underlying its use.

This study is particularly timely given the rapid evolution of the performance of the test and given that the political debate on the topic in Canada is gearing up. Two provinces and a territory have so far decided to offer public coverage of NIPT to women with pregnancies considered at high risk and discussions are ongoing in others. At the time that the survey ran, Ontario was the only province covering NIPT for women with high risk pregnancies. Thus, comparing attitudes in Ontario with the other provinces may be informative of how views regarding a medical technology are affected by shifting barriers to access.

A significant result of this study is the notable difference between the way in which pregnant women and couples prefer to see NIPT funded and the way healthcare professionals do. Namely, a majority of healthcare professionals thought that only high-risk pregnancies should be eligible for funding, while the majority of pregnant women and partners thought that all pregnancies should be eligible. It is important to remember that both women and partners, and healthcare professionals, received the same information regarding the performance and the limitations of NIPT (see Fig. 1 = informational sheet).

Furthermore, women and partners thought that their decision to test would be highly impacted by NIPT being accessible free of charge. Simultaneously, when asked whether they are concerned that NIPT being covered as part of routine prenatal care could lead to increased pressure on women to use it, the majority of pregnant women reported ‘no concern’ whatsoever and only a very small percentage (1.8%) reported being ‘very concerned’. These results may mean that the impact of public funding for NIPT on women’s decision-making would be rather autonomy-enhancing.

Nevertheless, it is important to consider the minority of Canadian women and couples who prefer not to screen for DS, and the concern that routinization of NIPT (i.e. the test being covered and offered routinely) may exacerbate current pressures to screen and/or terminate following a diagnosis, thereby restricting their reproductive autonomy [24–26]. Pregnant women from Ontario, the only province covering NIPT for high-risk pregnancies at the time of the study, were willing to pay significantly less than women from all other provinces. This was the only question where attitudes held by pregnant women in Ontario differed from the rest of Canada.

Healthcare professionals thought financial cost is an important barrier to accessing NIPT in Canada, and while they were more concerned than pregnant women about the potential for NIPT routinization to pressure women into screening, they were still largely unconcerned. Even so, although the groups seem to largely agree regarding how cost affects patients’ decision-making, healthcare professionals did not see the issue of barrier to access as justifying coverage of NIPT for all Canadian women. This may be due to healthcare professionals being more sensitive to issues of justice and prioritization, and their awareness that publicly funding a certain intervention means foregoing another. Or, it may be due to the fact that healthcare professionals have a deeper and more nuanced understanding of the limitations of NIPT as an emerging technology and its reliability as depending on the population in which it is performed (high versus low risk pregnancies). Given that views on public coverage are divided, it seems imperative to ensure transparency in how all available evidence regarding stakeholders’ preferences is taken into account in policy-making.

Another notable difference in the preferences of healthcare professionals and pregnant women and their partners related to how NIPT should be used. At the time of the survey, recommended practice was to offer NIPT as second-tier screening, i.e. to pregnant women at high risk for trisomy, and confirming any positive NIPT result with a diagnostic test. Half of surveyed healthcare professionals’ preferences were aligned with these professional guidelines, but a sizeable minority believed that NIPT ought to replace MSS as a first-tier screening technology. However, couples were unequivocally more interested in a broader use of NIPT than health professionals or than current policies recommend. While the performance of NIPT in low risk pregnancies has been the focus of much scientific debate [27, 28], the strong preference of women and partners for its use as a first-tier screen means that it may be of great value to them even at a time that its reliability in this population is still debated, so long as women are fully and clearly informed regarding the limitations of the test and the exact meaning of its results in their case.

It was surprising that 2.2% of healthcare professionals surveyed thought NIPT’s current performance justifies using it as a diagnostic test to replace amniocentesis, considering that some women may choose to terminate a pregnancy based on a positive result. Health professionals’ most preferred testing pathway, i.e. adding NIPT as a second-tier test, has been criticized as unnecessarily medicalizing pregnancy, although some have argued that the additional anxiety induced by adding another test may be alleviated by “the woman’s knowledge that she had followed a testing pathway designed to minimize risk to the pregnancy” [29]. To alleviate some of the additional anxiety, the ethically controversial solution of ‘reflex testing’ has been proposed [30], where two blood samples are taken during conventional serum screening, and if the first-tier screening result is “high risk”, the second blood sample is sent for NIPT in order to inform the woman of the result only once more reliable NIPT results are available. However, if this process is not reliably explained beforehand, this could result in decreased autonomy in reproductive decision-making in at least some cases, due to women receiving their NIPT results without appropriate consent [30].

Nearly 4/5 of the pregnant women, on the other hand, reported preferring replacing current screening with NIPT (NIPT as first-tier test), citing increased accuracy and earlier availability of results as the main reasons. Moreover, over 2/3 of pregnant women preferred NIPT to amniocentesis as a diagnostic test after current screening, disregarding the possibility of false positives and negatives, stating that avoiding the risk of miscarriage associated with amniocentesis is prospective parents’ most important consideration, corroborating the literature indicating the importance of avoiding risk to the fetus in women’s decision-making regarding their prenatal care [31–33].

Interestingly, reporting having been informed about NIPT by a genetic counsellor correlated with more frequent preference on the part of pregnant women for amniocentesis, a preference more aligned with current recommendations. Further research is warranted into possible causality behind this correlation. In particular, reporting having been informed by a genetic counsellor in this study also correlated with other demographic variables (namely having a child with DS, having a child with a disability, being low or high-risk for the current pregnancy, and having had a prenatal diagnosis in the past (*p* < 0.001); and to lesser extents, the age of the respondent, their province of residence, the way the current pregnancy was conceived, and having undergone prenatal screening in the past, as well as the kind of result of the said screen (*p* < 0.05)). However, could genetic counsellors be more cautious regarding the use of NIPT over current diagnostic tests due to their possibly more nuanced understanding of its limits? Could communicating with a genetic counsellor thus enhance reproductive autonomy? Depending on what future research concludes, important policy decisions might have to be made in Canada in relation to the fact that only 7.9% of the pregnant women in this survey were informed about current screening, NIPT or amniocentesis by genetic counsellors.

While 52.9% of pregnant women reported being interested in knowing whether their fetus has DS in order to “consider terminating the pregnancy if the baby was diagnosed with DS”, that leaves a sizable portion of the population testing for other reasons, medical or not [34]. Only 2.3% claim they do not want to know at all. As remarked by Nowotny, “worrying about risks is another way of coping with uncertainty. Unable to face the complexity that surrounds them, people take refuge in what is familiar. These are the risks they know. Worrying about them provides some comfort” [35]. It thus makes sense that so few people preferred amniocentesis. Why risk miscarriage if they might want to know “for knowing’s sake” only? In this context, NIPT can provide a measure of psychological tranquility, even if the result is positive.

## Limitations

Even though the respondents were chosen from a population that can most easily imagine themselves faced with the choices this study examines, i.e., pregnant women, their partners and health professionals dealing with pregnant women, it bears noting that stated hypothetical preferences do not always accurately predict what decisions the respondents would actually make in real-life situations. Our response rate is 47.1%, which is not that different from other similar studies [36]. It is possible that participants self-selected based on specific preferences that are not necessarily representative of the pregnant population in Canada as a whole. The choice of *p* < 0.001 as a threshold of statistical significance could have led to overlooking potentially valuable insights; however, in the interest of not overstating the findings, this more conservative choice was made by the research team. Additionally, although the aggregated results presented herein may be interpreted as if there is a monolithic group of Canadian pregnant women, it is important to note that Canadian pregnant women’s decision-making regarding NIPT can be differently affected by relevant policies and contexts that vary between regions.

## Conclusion

The key contributions of this study are three-fold. First, it reveals Canadian stakeholders’ preferences regarding public coverage of NIPT and their expected repercussions of universal coverage on women’s decision regarding testing, and draws attention to evidence of shifting attitudes towards coverage as policy evolves. Second, it reveals the contrasting testing preferences of expecting parents and health professionals. Third, it examines the factors that could potentially influence Canadians’ preferences.

## Additional Files

Additional file 1: Survey for Pregnant Women: blank questionnaire that was administered to pregnant women. (PDF 211 kb)

Additional file 2: Survey for Partners: blank questionnaire that was administered to pregnant women’s partners. (PDF 267 kb)

Additional file 3: Survey for Health Professionals: blank questionnaire that was administered to health professionals. (PDF 477 kb)

## Abbreviations

cfDNA: cell-free DNA
CHU: Centre Hospitalier et Universitaire (Hospital and University Centre)
CRCHU: Centre de Recherche du CHU (CHU Research Centre)
DS: Down syndrome
MSS: maternal serum screening
NIPT: Non-Invasive Prenatal Testing
PEGASUS: Personalized Genomics for prenatal Aneuploidy Screening Using maternal blood

## Footnotes

1 We are using the older term NIPT in order to stay consistent with the terminology as presented to study participants, even though terminology has shifted and much of the literature currently uses ‘cell-free DNA screening’ (or cfDNA screening).
