# Supplementary material for: The value of non-invasive prenatal testing: preferences of Canadian pregnant women, their partners, and health professionals regarding NIPT use and access
Source: BMC Pregnancy Childbirth. 2019 Jan 10;19:22. doi: 10.1186/s12884-018-2153-y (PMC6327577; doi:10.1186/s12884-018-2153-y)
Supplement: Supplementary file 2 — Survey for Partners: blank questionnaire that was administered to pregnant women’s partners. (PDF 267 kb) [file 12884_2018_2153_MOESM2_ESM.pdf]

4645

ANNN

Survey number (partners): [ ][ ][ ][ ]

SURVEY FOR PARTNERS

Please read the following before completing the survey:

STUDY DESCRIPTION
NIPT (Non-Invasive Prenatal Testing) is a new technology that tests the risk for Down syndrome. The purpose of this survey is to learn what pregnant women and their partners know about NIPT and to learn about their views regarding its use. This survey is part of a larger study on NIPT, called PEGASUS, see: http://pegasus-pegase.ca/.

BENEFITS AND RISKS
Participating in the study will not provide you with any immediate or specific benefit, but it will allow you to contribute to the advancement of knowledge about NIPT and this may help other women who use this test in the future. The survey does not pose any risk and its results will be kept confidential. You are free to withdraw from this study at any time.

CONSENT
By completing and returning this survey, you consent to participate in this part of the PEGASUS study and authorize Dr. Vardit Ravitsky and her colleagues to analyze the content of the completed survey. Completing this survey can take about 20 minutes.

CONFIDENTIALITY
This survey is coded and you do not have to sign your name on it. All information obtained in connection with this survey will be kept confidential. Access to this survey will be restricted to the members of the research team, for the duration of the study. The surveys will be kept in a secure place, under lock and key, for a maximum of 10 years after the project ends. The results of the study may be published, but no identifiable information will ever be disclosed.

CONTACT PERSONS
For further information regarding this project, you are welcome at any time to contact Dr. Vardit Ravitsky at (514) 343-6111 extension 3375 or at vardit.ravitsky@umontreal.ca.

INSTRUCTIONS
Please answer directly on the survey. If you change your mind, cross out your first mark. When you are finished, please seal it in the attached envelope and hand it in or return it in the pre-addressed envelope.

If you prefer to complete this survey online, you can find it at: http://nipt.hostedincanadasurveys.ca/s1

We thank you for participating.

© Vardit Ravitsky, Anne-Marie Laberge

4645

PART 1: WHAT DO YOU KNOW ABOUT DOWN SYNDROME (DS)?

1. **Before** today, have you ever heard of:
(**PLEASE CHECK ONE ANSWER FOR EACH STATEMENT**)

		Yes	No
a.	Down syndrome	☐	☐
b.	Trisomy 21	☐	☐
c.	Prenatal screening for Down syndrome	☐	☐
d.	Noninvasive prenatal testing (NIPT)	☐	☐
e.	Amniocentesis	☐	☐

2. The next question is about Down syndrome (DS). Do you think these statements are true or false?
(**PLEASE CHECK ONE ANSWER FOR EACH STATEMENT**)

		True	False
a.	Other people can tell when a child has DS	☐	☐
b.	All individuals with DS have some kind of intellectual disability	☐	☐
c.	People with DS can live until at least their 50's-60's	☐	☐
d.	Intellectual disability is the only health issue related to DS	☐	☐
e.	DS can be caused by a woman's unhealthy lifestyle	☐	☐
f.	A woman has a risk of having a baby with DS **only if** somebody in her family has DS	☐	☐

3. The next question is about the different options available to pregnant women who want to know more about the risk of Down syndrome (DS) for their pregnancy. Do you think these statements are true or false?
(**PLEASE CHECK ONE ANSWER FOR EACH STATEMENT**)

		True	False
a.	All babies with DS show signs of DS on ultrasound	☐	☐
b.	Current screening can be used to diagnose with certainty a baby with DS	☐	☐
c.	Amniocentesis can be used to diagnose with certainty a baby with DS	☐	☐
d.	NIPT can be used to diagnose with certainty a baby with DS	☐	☐
e.	Amniocentesis can predict the severity of the symptoms of DS	☐	☐
f.	There is an increased risk of miscarriage (losing the pregnancy) with amniocentesis	☐	☐
g.	There is no increased risk of miscarriage (losing the pregnancy) with NIPT	☐	☐

© Vardit Ravitsky, Anne-Marie Laberge

INFORMATIONAL SHEET

Down syndrome (DS) is a genetic condition caused by the presence of an extra chromosome 21 (also called 'trisomy 21') which affects 1 in 770 newborns. Individuals with DS usually share physical features that are characteristic of DS. All have some degree of intellectual disability, which varies from person to person; their development is slower than other kids, but they will eventually learn to walk, talk, and dress themselves. Most children attend their neighborhood schools, some in regular classes and others in special education classes. Some children have more significant needs and require a more specialized program. Many adults with DS are capable of working in the community, but some require a more structured environment. Many will also have other health problems (for example heart defects). 99% of cases of DS are not inherited from the parents; it usually occurs by chance.

There are ways to check during pregnancy if there is a possibility that the baby has DS:

	**MATERNAL SERUM SCREENING (MSS or 'current screening')**	**AMNIOCENTESIS**	**NIPT**
**Description of the procedure**	• Checks the **level of risk** for DS<br>• Measures the **level of hormones** produced by the baby or placenta that end up in the mother's blood<br>• Includes **one or two blood draws** from the mother<br>• Where available, an ultrasound is done early in the pregnancy to measure nuchal translucency (level of fluid at the nape of the baby's neck)	• Medical procedure that can **confirm DS** during the pregnancy<br>• Allows checking the **number and appropriate structure of all chromosomes** in the baby's cells<br>• Requires **inserting a thin needle into the uterus** – through the mother's abdomen - to extract amniotic fluid (fluid in which the baby floats in the mother's womb)	• Checks the **level of risk** for DS<br>• Analyses the **baby's DNA** that is floating in the mother's blood<br>• Includes **one blood draw** from the mother
**Timing: When in pregnancy**	• 1st blood draw: usually between the **10th and 13th week** of pregnancy<br>• 2nd blood draw: usually between the **15^{h} and 16th** week of pregnancy<br>• Results can be available between the **16th and 17th** week of pregnancy	• Available from the **15th week** of pregnancy<br>• Results can be available between the **17th and 19th week** of pregnancy	• Available as of the **10th week** of pregnancy<br>• Results can be available between the **11th-12th week** of pregnancy
**Risk to pregnancy**	**No increased risk of miscarriage**	**Risk of miscarriage** around 1 in 200 (0.5%)	**No increased risk of miscarriage**
**Accuracy**	Detects between 77% and 88% of DS cases (supported by a lot of evidence)	100% accurate in detecting DS (supported by a lot of evidence)	98% accurate (or more) for DS in women who are considered "high risk" based on MSS (supported by some evidence)
**Type of test**	Screening	Diagnostic	Screening
**What it detects**	• Down syndrome<br>• Trisomy 18<br>• Neural tube defects (e.g. spina bifida)<br>• Possible pregnancy complications	• Down syndrome<br>• Trisomy 13<br>• Trisomy 18<br>• Other chromosome anomalies<br>• Neural tube defects (e.g. spina bifida)<br>• Sex of the baby	• Down syndrome<br>• Trisomy 13<br>• Trisomy 18<br>• Sex of the baby
**Possible outcome**	• The test can predict that the pregnancy is at:<br>→ **Low risk** for DS (less than 1/200 – 1/300) so no further test is recommended<br>→ **High risk** for DS (higher than 1/200 – 1/300)<br>• **Amniocentesis is** offered to check if the baby actually has DS or other abnormalities detectable by chromosome analysis.	• **Normal result**: the baby does not have DS and has normal chromosomes.<br>• **Abnormal result**: the baby has DS or has another significant chromosome abnormality. In this case, the parents can choose to:<br>→ **continue the pregnancy**<br>→ **stop the pregnancy**	• The test can predict that the pregnancy is at:<br>→ **Very low risk** for DS - so no further test is recommended<br>→ **Very high risk** for DS<br>• **Amniocentesis is recommended** to confirm that the baby has DS

Note: NIPT is not yet considered as a first-tier screening test (i.e. that could replace serum screening); the current available evidence supports its use as a second tier screening test – after a positive serum screening and before an amniocentesis.

Last update: July 15, 2014
© PEGASUS: Vardit Ravitsky, Anne-Marie Laberge

CHU de Québec

PEGASUS PEGASE

4. Information about a baby having Down syndrome (DS) can have different uses for different people. Why are you interested in knowing whether your baby has DS?
(PLEASE CHECK ONE ANSWER)

- ☐ I want to know in advance to prepare for the birth of a baby with DS if baby is diagnosed with DS
- ☐ I would consider terminating the pregnancy if the baby was diagnosed with DS
- ☐ I'm unsure
- ☐ I don't want to know but my partner does

Other:

PART 2: INFORMED CONSENT

5. When would be the **best** time for you to receive the following information:
(PLEASE CHECK ONE ANSWER PER STATEMENT)

		Early prenatal appointment ahead of time of NIPT	Same day as blood test for NIPT	When your partner gets NIPT results	Not interested in this information
**a.**	What is Down syndrome (DS)	☐	☐	☐	☐
**b.**	How good is NIPT in detecting DS	☐	☐	☐	☐
**c.**	What the test can and cannot tell	☐	☐	☐	☐
**d.**	What is the chance that you can have a baby with DS (according to family history, your partner's age, etc.)	☐	☐	☐	☐
**e.**	What are the possible results (high vs. low risk for DS)	☐	☐	☐	☐
**f.**	What are the options if the result is positive	☐	☐	☐	☐
**g.**	What are the available resources for families who have children with DS	☐	☐	☐	☐

Other:

6. Do you think it is important to get written consent for NIPT considering there is no risk of miscarriage?
(PLEASE CHECK ONE ANSWER ONLY)

☐ Yes ☐ No ☐ I don't know ☐ Other: ________________________________

© Vardit Ravitsky, Anne-Marie Laberge

7. Please rank in order of importance the people you would like to discuss NIPT with:
(1= YOUR FIRST CHOICE, 6 = YOUR LAST CHOICE)

☐ Family physician

☐ Obstetrician/Gynecologist

☐ Genetics specialist

☐ Nurse

☐ Midwife

☐ Other:____

8. How would you like to be informed about NIPT?
(PLEASE CHECK ALL THAT APPLY)

☐ Brochures ☐ Video ☐ Discussion with prenatal caregiver
☐ Website ☐ Group meetings ☐ Other: _____________________________

9. How much time would you want to make a decision about NIPT?
(PLEASE CHECK ONE ANSWER ONLY)

☐ I would make a decision during the appointment when my partner is offered the test.
☐ I would want a few days to think about it.
☐ Not sure.

10. What would be the most appropriate way for you to receive the results of NIPT?
(PLEASE CHECK ONE ANSWER FOR EACH STATEMENT)

		In person	By phone	By mail, fax or email	Through a secured website	I don't care
a.	If NIPT result is LOW risk of DS	☐	☐	☐	☐	☐
b.	If NIPT result is HIGH risk of DS	☐	☐	☐	☐	☐

**Other:**

© Vardit Ravitsky, Anne-Marie Laberge

PART 3: HOW SHOULD NIPT BE USED?

11. George and Melissa are expecting a baby. Melissa is 40 years old and is 10 weeks pregnant. They are meeting her doctor for her first prenatal visit. During their visit, the doctor explains that:

- *Current screening* can assess the risk that the baby might have Down syndrome (DS). It can also tell her the risk of neural tube defects (such as spina bifida) and give her information about the risk of complications during pregnancy and labor. However, current screening detects only about 80% of cases of DS and Melissa will only get her results after the 15th week of pregnancy.
- *NIPT* is available earlier in the pregnancy (around 10 weeks) and will detect 99% of cases of DS. NIPT can also predict the risk that the baby might have a sex chromosome disorder (where the baby has an extra or missing sex chromosome). However, it cannot tell whether the baby might have a neural tube defect or about the risk of pregnancy and labour complications.

11.1. If you were George and you had to choose between current screening and NIPT as a first step, which test would you choose for the pregnancy?
(PLEASE CHECK ONE ANSWER ONLY)

☐ Current screening

☐ NIPT

☐ I would not want any testing (please skip the next question and go to question 12)

11.2. How did the following reasons influence your decision?
(PLEASE CIRCLE ONE ANSWER FOR EACH STATEMENT)

		Does not influence		Somewhat influences		Strongly influences
**a.**	Current screening estimates the risk that the baby has neural tube defects and NIPT doesn't	1	2	3	4	5
**b.**	Current screening can indicate the possibility of pregnancy and labor complications and NIPT cannot	1	2	3	4	5
**c.**	NIPT is much more accurate than current screening in assessing the risk of DS	1	2	3	4	5
**d.**	Results of NIPT can be available earlier in the pregnancy than the result of current screening	1	2	3	4	5

Other:

© Vardit Ravitsky, Anne-Marie Laberge

12. Robert and Carolyn are expecting a baby. Carolyn is 40 years old and is 16 weeks pregnant. They are meeting her doctor for a prenatal visit. The pregnancy is at high risk for Down syndrome (meaning that there is more than 1 in 300 chance that her baby has DS) based on current screening.

They are referred to a genetic specialist, who explains that there are further tests available:

- *Amniocentesis* is an invasive procedure that is available from the 15th week of pregnancy. Because a needle would be inserted into Carolyn's womb, there is an increased risk of miscarriage due to the procedure (0.25 to 0.5%). This test is practically 100% accurate, and makes it possible to check the number of all of the baby's chromosomes.
- *NIPT* is done earlier in the pregnancy (about 10 weeks). Because it only requires a blood draw, there is no increased risk of miscarriage. However, NIPT cannot check all of the baby's chromosomes. NIPT is a very accurate test (will detect about 99% of cases of DS), but is not as accurate as amniocentesis.

12.1. If you were Robert and you had to choose between amniocentesis and NIPT as a diagnostic test after current screening, which test would you choose for the pregnancy?
(PLEASE CHECK ONE ANSWER ONLY)

☐ Amniocentesis

☐ NIPT

☐ I would not want any testing (please skip the next question and go to question 13)

12.2. How did the following reasons influence your decision?
(PLEASE CIRCLE ONE ANSWER FOR EACH STATEMENT)

		Does not influence		Somewhat influences		Strongly influences
**a.**	Amniocentesis is more accurate than NIPT	1	2	3	4	5
**b.**	Amniocentesis gives more information about possible chromosome anomalies than NIPT	1	2	3	4	5
**c.**	With NIPT there is no increased risk of miscarriage	1	2	3	4	5
**d.**	NIPT tests for the common chromosome disorders (like DS), which is all I need to know	1	2	3	4	5
**e.**	NIPT is more convenient than amniocentesis. (only requires a blood draw)	1	2	3	4	5

Other:

© Vardit Ravitsky, Anne-Marie Laberge

4645

13. Right now, NIPT is not covered by the health care system in most provinces and territories. How much would you be willing to pay for NIPT?
(PLEASE CHECK ONE ANSWER ONLY)

☐ 0$ ☐ 100$ to 499$ ☐ 1000$ to 4999$
☐ 1$ to 99$ ☐ 500$ to 999$ ☐ More than 5000$

14. Who do you think should have access to NIPT free of charge?
(PLEASE CHECK ONE ANSWER ONLY)

☐ All women ☐ Low risk women only ☐ Other: ____________________
☐ High risk women only ☐ Nobody (you should pay for the test)

15. If NIPT were free of charge, how do you think that would impact your decision to have the test?
(PLEASE CIRCLE ONE ANSWER)

No impact		Some impact		A lot of impact
1	2	3	4	5

PART 4: DECISION MAKING AND THE INVOLVEMENT OF OTHERS

16. Please rank in order of importance the input of the following people in your desire to do NIPT for the pregnancy:
(1 = THE MOST IMPORTANT; 5/6 = THE LEAST IMPORTANT)

☐ Partner

☐ Family

☐ Friends and colleagues

☐ Your own prenatal care professional

☐ A specialist (medical geneticist, obstetrician)

☐ Other: ____

© Vardit Ravitsky, Anne-Marie Laberge

4645

17. How much input do you feel a partner **should have** on a woman's decision about one of these following tests?
(PLEASE CIRCLE ONE ANSWER FOR EACH STATEMENT)

		No input		Some input		A lot of input
**a.**	Current screening	1	2	3	4	5
**b.**	Amniocentesis	1	2	3	4	5
**c.**	NIPT	1	2	3	4	5

18. Do you feel that your opinion is **actually** taken into account by your **partner** when deciding about one of the following tests?
(PLEASE CIRCLE ONE ANSWER FOR EACH STATEMENT)

		Not at all		Somewhat		Very much
**a.**	Current screening	1	2	3	4	5
**b.**	Amniocentesis	1	2	3	4	5
**c.**	NIPT	1	2	3	4	5

19. Do you feel that your opinion as a partner is taken into account by **healthcare professionals** when deciding about one of the following tests?
(PLEASE CIRCLE ONE ANSWER FOR EACH STATEMENT)

		Not at all		Somewhat		Very much
**a.**	Current screening	1	2	3	4	5
**b.**	Amniocentesis	1	2	3	4	5
**c.**	NIPT	1	2	3	4	5

20. If your partner disagreed with your desire to do or not NIPT, what impact would that have on your view?
(PLEASE CIRCLE ONE ANSWER ONLY)

No impact		Some impact		A big impact
1	2	3	4	5

Any other comments on the impact a disagreement would have on your decision:

© Vardit Ravitsky, Anne-Marie Laberge

PART 5: SOCIAL IMPACT OF NIPT

21. Provincial health care systems cover routine prenatal care. Right now, NIPT is not part of routine prenatal care in most provinces and territories. If NIPT were covered as part of routine prenatal care, which of the following outcomes would be of concern to you?

(PLEASE CIRCLE ONE ANSWER FOR EACH STATEMENT)

		Not concerned		Somewhat concerned		Very concerned
**a.**	Increased pressure on women to use NIPT	1	2	3	4	5
**b.**	Increased use of NIPT leading to increased pressure to terminate if the baby has Down syndrome (DS)	1	2	3	4	5
**c.**	Increased availability of NIPT making people less willing to accept children with disabilities	1	2	3	4	5
**d.**	Decrease of the population of people with DS	1	2	3	4	5
**e.**	Reduction in resources available for people with DS and their families	1	2	3	4	5
**f.**	Negative impact on individuals with DS and their families (stigma, discrimination)	1	2	3	4	5

Other:

22. If NIPT became part of routine tests offered during the pregnancy, how likely is it that:

(PLEASE CIRCLE ONE ANSWER FOR EACH STATEMENT)

		No pressure		Some pressure		A lot of pressure
**a.**	**your partner** would feel pressure to take the test because it would be routine	1	2	3	4	5
**b.**	**you** would feel pressure to take the test for the pregnancy because it would be routine	1	2	3	4	5

© Vardit Ravitsky, Anne-Marie Laberge

PART 6: FUTURE USES OF NIPT

23. In the future, NIPT may be able to test for many genetic conditions. How interested would you be in NIPT for the following conditions:

(PLEASE CIRCLE ONE ANSWER FOR EACH STATEMENT)

		Not interested		Somewhat interested		Very interested
**a.**	Inherited disorders (such as Tay-Sachs, cystic fibrosis, sickle cell disease, Gaucher disease)	1	2	3	4	5
**b.**	Paternity testing	1	2	3	4	5
**c.**	Physical and behavioural attributes (eye colour, intelligence, sexual orientation)	1	2	3	4	5
**d.**	Predisposition to childhood-onset diseases (autism, leukemia)	1	2	3	4	5
**e.**	Predisposition to late-onset diseases (heart problems, Alzheimer's disease, cancer)	1	2	3	4	5
**f.**	Predisposition to mental disorders (schizophrenia, bipolar disease)	1	2	3	4	5

Other:

PART 7: ABOUT YOURSELF

24. Your birth date (Month/Year): MM/AAAA ☐☐ / ☐☐☐☐

25. Your gender: _______________

26. Your country of birth: ____

27. What language do you mostly speak **at home**?

☐ English ☐ French ☐ Other: ________________________

© Vardit Ravitsky, Anne-Marie Laberge

28. In which province or territory do you **currently live**?
(PLEASE CHECK ONE ANSWER ONLY)

☐ Alberta
☐ New Brunswick
☐ Northwest Territories
☐ Ontario
☐ Saskatchewan
☐ British Columbia
☐ Newfoundland and Labrador
☐ Nunavik
☐ Prince-Edward-Island
☐ Yukon
☐ Manitoba
☐ Nova Scotia
☐ Nunavut
☐ Quebec

29. What is your race/ethnicity?
(PLEASE CHECK ALL THAT APPLY)

☐ Caucasian
☐ African descent
☐ North American Aboriginal (First Nation, Inuit, Metis)
☐ Latin American
☐ Middle Eastern
☐ Other: _______________________________
☐ Asian

30. What is your religious/cultural background?
(PLEASE CHECK ALL THAT APPLY)

☐ Buddhist
☐ Christian
☐ Muslim
☐ No religious affiliation
☐ Catholic
☐ Jewish
☐ Protestant
☐ Other: _____________________
☐ Catholic Orthodox
☐ Hindu
☐ Sikh

31. How important is religion / spirituality in your life?
(PLEASE CIRCLE ONE ANSWER ONLY)

**Not important**		**Somewhat important**		**Very important**
1	2	3	4	5

32. What is the highest level of school you *completed*?
(PLEASE CHECK ONE ANSWER ONLY)

☐ Elementary school
☐ High school
☐ University
☐ Trades/Apprenticeship
☐ College
☐ Other : _______________________

© Vardit Ravitsky, Anne-Marie Laberge

33. Are you a healthcare professional?
(PLEASE CHECK ONE ANSWER ONLY)

☐ Yes → **33.1.** If yes, what profession?
____

☐ No

34. What is your relationship status?
(PLEASE CHECK ONE ANSWER ONLY)

☐ Married ☐ Single ☐ Widowed
☐ Common Law ☐ Divorced/separated ☐ Other : ________________________

35. Do you have children?
(PLEASE CHECK ONE ANSWER ONLY)

☐ Yes ☐ No

36. Do you have a child with Down syndrome?
(PLEASE CHECK ONE ANSWER ONLY)

☐ Yes ☐ No

37. Do you have a child with a physical or intellectual disability?
(PLEASE CHECK ONE ANSWER ONLY)

☐ Yes ☐ No

38. Does anyone close to you have a child with Down syndrome?
(PLEASE CHECK ONE ANSWER ONLY)

☐ Yes ☐ No

© Vardit Ravitsky, Anne-Marie Laberge

CHU de Québec

PEGASUS PEGASE

39. For this pregnancy, is your partner considered:
(PLEASE CHECK ONE ANSWER ONLY)

- ☐ Low/average risk for Down syndrome
- ☐ High risk for Down syndrome
- ☐ Unsure

40. For this pregnancy, who provided you information about current screening, amniocentesis and/or NIPT?
(PLEASE CHECK ALL THAT APPLY)

- ☐ Family physician
- ☐ Obstetrician/Gynecologist
- ☐ Medical geneticist
- ☐ Genetic Counsellor
- ☐ Nurse
- ☐ Midwife
- ☐ Partner
- ☐ Other: _______________

41. This pregnancy was conceived:
(PLEASE CHECK ONE ANSWER ONLY)

- ☐ Naturally
- ☐ by *in vitro* fertilization (IVF)
- ☐ with assisted reproductive technology (ART), other than IVF
- ☐ Other : _________________________

42. Has your partner had **prenatal screening** in a **previous pregnancy**?
(PLEASE CHECK ONE ANSWER ONLY)

- ☐ Yes → **If yes**, what was the result of the prenatal screening?
  (PLEASE CHECK ONE ANSWER ONLY)
  - ☐ High risk for Trisomy (e.g. Down Syndrome, trisomy 13, trisomy 18)
  - ☐ High risk for a Neural Tube Defect (e.g. spina bifida)
  - ☐ Low or average risk
  - ☐ Unsure
- ☐ No
- ☐ Unsure

43. Has your partner had **prenatal diagnosis** (chorionic villus sampling or amniocentesis) in a **previous pregnancy**?
(PLEASE CHECK ONE ANSWER ONLY)

- ☐ Yes → **If yes**, what was the result of the prenatal diagnosis?
  (PLEASE CHECK ONE ANSWER ONLY)
  - ☐ Diagnosis of a genetic condition (e.g. cystic fibrosis, Down syndrome)
  - ☐ Normal result (no genetic condition detected)
  - ☐ Other: ____
- ☐ No
- ☐ Unsure

© Vardit Ravitsky, Anne-Marie Laberge

Thank you for completing this survey.

If you have any additional comments or thoughts, please write them below.

© Vardit Ravitsky, Anne-Marie Laberge
