# Supplementary material for: The value of non-invasive prenatal testing: preferences of Canadian pregnant women, their partners, and health professionals regarding NIPT use and access
Source: BMC Pregnancy Childbirth. 2019 Jan 10;19:22. doi: 10.1186/s12884-018-2153-y (PMC6327577; doi:10.1186/s12884-018-2153-y)
Supplement: Supplementary file 3 — Survey for Health Professionals: blank questionnaire that was administered to health professionals. (PDF 477 kb) [file 12884_2018_2153_MOESM3_ESM.pdf]

SURVEY FOR HEALTH PROFESSIONALS

Please read the following instructions before completing the questionnaire:

STUDY DESCRIPTION
NIPT (Non-Invasive Prenatal Testing) is a new technology used in high-risk pregnancies for detecting Down syndrome and other conditions. We are trying to learn what health professionals know about NIPT and what their perceptions and attitudes are regarding its clinical implementation and use. This questionnaire is part of a larger study on NIPT, called PEGASUS, see: http://pegasus-pegase.ca/.

CONSENT
By completing and returning this questionnaire, you consent to participate in this part of the PEGASUS study and authorize Dr. Vardit Ravitsky and her colleagues to analyze the content of the completed questionnaire. Completing this survey can take about 15 minutes.

CONFIDENTIALITY
This questionnaire is anonymous. All information obtained in connection with this questionnaire will be kept confidential. Access to this questionnaire will be restricted to the members of the research team, for the duration of the study. The questionnaires will be kept in a secure place, under lock and key, for a maximum of 10 years after the project ends. The results of the study may be published, but no identifiable information will ever be disclosed.

CONTACT PERSONS
For further information regarding this project, you are welcome at any time to contact Dr. Vardit Ravitsky at (514) 343-6111 extension 3375 or at vardit.ravitsky@umontreal.ca.

INSTRUCTIONS
Please answer directly on the questionnaire. When you are finished, please seal it in the attached envelope and hand it in or return it in the pre-addressed envelope.

If you prefer to complete this questionnaire online, you can find it at:
http://nipt.hostedincanadasurveys.ca/index.php/658186/

We thank you for participating.

© Vardit Ravitsky and Anne-Marie Laberge

PART 1: WHAT DO YOU KNOW ABOUT NIPT?

1. Do you think these statements are true or false?
(PLEASE CHECK ONE ANSWER FOR EACH STATEMENT)

		True	False
**a.**	NIPT is currently accepted as a diagnostic test for Down syndrome (DS)	☐	☐
**b.**	Professional guidelines (e.g. SOGC) recommend that NIPT be offered to all pregnant women	☐	☐
**c.**	It is currently recommended to confirm a positive result of NIPT with invasive testing	☐	☐
**d.**	NIPT has a detection rate of almost 100% for DS in high risk pregnancies	☐	☐
**e.**	NIPT can estimate the risk for neural tube defects, like current maternal serum screening	☐	☐
**f.**	NIPT can be used for sex determination	☐	☐
**g.**	NIPT is offered only after the 15th gestational week	☐	☐

2. How comfortable are you in describing the following information about Down syndrome (DS) and NIPT to patients?
(PLEASE CHECK ONE ANSWER FOR EACH STATEMENT)

		Not comfortable		Somewhat comfortable		Very comfortable
**a.**	Clinical description of DS (phenotype, variability, prognosis)	1	2	3	4	5
**b.**	Accuracy and limits of NIPT (false-positives, false-negatives, range of conditions tested)	1	2	3	4	5
**c.**	Patient's personal risk assessment (according to family history, age, previous pregnancy history)	1	2	3	4	5
**d.**	Options available if NIPT comes back positive for DS	1	2	3	4	5
**e.**	Resources available for families of children with DS	1	2	3	4	5

The following sections contain information on NIPT. Please do not change your previous answers based on the information provided in the next sections. Since this is a new test, we want to know what professionals know about NIPT before answering the survey.
Thank you!

© Vardit Ravitsky and Anne-Marie Laberge

PART 2: FEATURES OF NIPT

NONINVASIVE PRENATAL TESTING (NIPT) can detect if a pregnancy is at a higher risk for Down syndrome (DS) and requires only a blood draw from the pregnant woman as early as 10 weeks gestation. There is no risk of miscarriage and it can predict with over 99% accuracy if the fetus has DS. However, it is not a diagnostic test at this time and amniocentesis should be done for confirmation. NIPT can detect higher risk of trisomy 13 and 18, but with less accuracy. It can also confirm sex, but not whether the baby has neural tube defects. Please see a comparative table of current tests (appendix).

3. How important would the following reasons be in your decision to offer NIPT (in general, not to a specific patient)?
(PLEASE CIRCLE ONE ANSWER FOR EACH STATEMENT)

		Not important		Somewhat important		Very important
**a.**	Absence of miscarriage risk	1	2	3	4	5
**b.**	Better accuracy than current screening	1	2	3	4	5
**c.**	Ease of use	1	2	3	4	5
**d.**	Recommendation of professional guidelines	1	2	3	4	5
**e.**	Clinical validity	1	2	3	4	5

Other:

4. When offering NIPT for DS, how important do you think it is to discuss the following information with your patient?
(PLEASE CIRCLE ONE ANSWER FOR EACH STATEMENT)

		Not important		Somewhat important		Very important
**a.**	Clinical description of DS (phenotype, variability, prognosis)	1	2	3	4	5
**b.**	Accuracy and limits of NIPT (false-positives, false-negatives, range of conditions tested)	1	2	3	4	5
**c.**	Patient's personal risk assessment (according to family history, age, previous pregnancy history)	1	2	3	4	5
**d.**	Options available if NIPT comes back positive for DS	1	2	3	4	5
**e.**	Resources available for families of children with DS	1	2	3	4	5

Other:

5. When do you feel is the **best** time to discuss with your patients the following features of NIPT?
(PLEASE CHECK ONE ANSWER FOR EACH STATEMENT)

		First prenatal appointment ahead of time of NIPT	Same day as blood draw for NIPT	When giving NIPT results
**a.**	Clinical description of DS (phenotype, variability, prognosis)	☐	☐	☐
**b.**	Accuracy and limits of NIPT (false-positives, false-negatives, range of conditions tested)	☐	☐	☐
**c.**	Patient's personal risk assessment (according to family history, age, previous pregnancy history)	☐	☐	☐
**d.**	Options available if NIPT comes back positive for DS	☐	☐	☐
**e.**	Resources available for families of children with DS	☐	☐	☐

Other:

© Vardit Ravitsky and Anne-Marie Laberge

PART 3: HOW SHOULD WE USE NIPT?

6. Do you think it is important to get written consent for NIPT?
(PLEASE CHECK ONE ANSWER ONLY)

☐ Yes ☐ No ☐ I'm not sure

Why? ____

____

7. There are different ways that NIPT can be used. Which one do you think is currently the most appropriate approach?
(PLEASE CHECK ONE ANSWER)

☐ Current screening using ultrasound and/or MSS, followed by NIPT as a second-tier screening (confirmed with amniocentesis)
☐ NIPT as first-tier screening (replacing MSS), confirmed with amniocentesis
☐ NIPT as a diagnostic test (without confirmation by amniocentesis), then availability of pregnancy termination if NIPT result is positive
☐ Other: ____

8. What following reasons would make you **not offer** NIPT to a specific patient?
(PLEASE CHECK ALL THAT APPLY)

☐ My patient does not want to know whether the fetus has Down syndrome (DS)
☐ There is insufficient clinical data on NIPT
☐ I am not comfortable explaining the test
☐ My patient and/or her partner have no family history of DS
☐ My patient would have to pay for the test
☐ Other: ______________________________

9. Which of the following reasons would influence your decision **to offer** NIPT to a specific patient?
(PLEASE CHECK ALL THAT APPLY)

☐ The test is recommended by professional organizations (SOGC, CCMG, ACMG)
☐ My patient asks for the test
☐ My patient is at a higher risk of having a child with DS
☐ My patient or her partner has a family history of DS
☐ NIPT would allow my patient to find out early in the pregnancy whether the fetus has DS or not
☐ If the cost of the test were covered
☐ Other: ______________________________

10. NIPT currently costs about 500-800$ in some private clinics. Who do you think should have access to NIPT free of charge?
(PLEASE CHECK ONE ANSWER ONLY)

☐ All women ☐ Low risk women only ☐ Other: ____________
☐ High risk women only ☐ Nobody (women should pay for it)

© Vardit Ravitsky and Anne-Marie Laberge

11. To what degree do you believe that the following features are barriers to clinical implementation of NIPT?
(PLEASE CIRCLE ONE ANSWER FOR EACH STATEMENT)

		Not a barrier		Somewhat of a barrier		Definite barrier
**a.**	Lack of coverage for the test (generally not reimbursed)	1	2	3	4	5
**b.**	Lack of knowledge by health professionals	1	2	3	4	5
**c.**	Lack of interest by the government	1	2	3	4	5
**d.**	Lack of interest by pregnant women and their partners	1	2	3	4	5
**e.**	Lack of resources (qualified lab personal, qualified labs)	1	2	3	4	5
**f.**	Lack of clinical validation studies	1	2	3	4	5
**g.**	Lack of equal access to the test	1	2	3	4	5

Other:

12. What would be the best way to inform health professionals about NIPT?
(PLEASE RANK: 1= YOUR FIRST CHOICE, 5/6 = YOUR LAST CHOICE)

- ☐ Professional guidelines
- ☐ Staff meetings
- ☐ Conferences
- ☐ Other: ____
- ☐ Journal clubs
- ☐ Ground rounds

PART 4: SOCIAL IMPACT OF NIPT

13. If NIPT became part of routine tests offered during pregnancy and covered by the healthcare system, do you think women would feel pressure to take it?
(PLEASE CIRCLE ONE ANSWER)

No pressure		Some pressure		A lot of pressure
1	2	3	4	5

14. Provincial health care systems cover routine prenatal care. Right now, NIPT is not part of routine prenatal care in most provinces and territories. If NIPT were covered as part of routine prenatal care, which of the following outcomes would be of concern to you?
(PLEASE CIRCLE ONE ANSWER FOR EACH STATEMENT)

		Not concerned		Somewhat concerned		Very concerned
**a.**	Increased pressure on women to use NIPT	1	2	3	4	5
**b.**	Increased use of NIPT leading to increased pressure to terminate if the baby has Down syndrome (DS)	1	2	3	4	5
**c.**	Increased availability of NIPT making people less willing to accept children with disabilities	1	2	3	4	5
**d.**	Decrease of the population of people with DS	1	2	3	4	5
**e.**	Reduction in resources available for people with DS and their families	1	2	3	4	5
**f.**	Negative impact on individuals with DS and their families (stigma, discrimination)	1	2	3	4	5

Other:

© Vardit Ravitsky and Anne-Marie Laberge

PART 5: FUTURE USES OF NIPT

15. In the **future**, NIPT may become a very reliable predictor of many genetic conditions. Are you in favour of NIPT being available for the following conditions:
(PLEASE CIRCLE ONE ANSWER FOR EACH STATEMENT)

		Not in favour		Somewhat in favour		In favour
**a.**	Inherited disorders (Tay-Sachs, cystic fibrosis, sickle cell disease, Gaucher disease)	1	2	3	4	5
**b.**	Paternity testing	1	2	3	4	5
**c.**	Physical and behavioural attributes (eye colour, intelligence, sexual orientation)	1	2	3	4	5
**d.**	Predisposition to childhood-onset diseases (autism, leukemia)	1	2	3	4	5
**e.**	Predisposition to late-onset diseases (heart conditions, Alzheimer's disease, cancer)	1	2	3	4	5
**f.**	Predisposition to mental disorders (schizophrenia, bipolar disease)	1	2	3	4	5

Other:

16. Technology today allows us to look for other chromosomal anomalies, including microdeletions and microduplications, using chromosomal microarrays or comparative genomic hybridization. How useful do you think it would be to perform such tests through NIPT in low-risk women? ?
(PLEASE CIRCLE ONE ANSWER)

Not useful		Somewhat useful		Very useful
1	2	3	4	5

PART 6: ABOUT YOURSELF

17. Your age: _____________

18. Your gender: ____________

19. What is your field of practice?
(PLEASE CHECK ONE ANSWER)

- ☐ General Practitioner
- ☐ Obstetrician/Gynecologist
- ☐ Genetic Counselor
- ☐ Midwife
- ☐ Pediatrician
- ☐ Clinical geneticist
- ☐ Nurse
- ☐ Other: ____________________

20. Years of practice: ____________________

21. In which province or territory are you **currently** practicing?
(PLEASE CHECK ONE ANSWER)

- ☐ Alberta
- ☐ New Brunswick
- ☐ Northwest Territories
- ☐ Ontario
- ☐ Saskatchewan
- ☐ British Columbia
- ☐ Newfoundland and Labrador
- ☐ Nunavik
- ☐ Prince-Edward-Island
- ☐ Yukon
- ☐ Manitoba
- ☐ Nova Scotia
- ☐ Nunavut
- ☐ Quebec

22. What is your **main** field of practice?
(PLEASE CHECK ONE ANSWER)

- ☐ Private practice
- ☐ Public hospital
- ☐ Other: _______________
- ☐ Research hospital
- ☐ Public health organization

© Vardit Ravitsky and Anne-Marie Laberge

23. Number of years of experience you have working in a prenatal setting: ________________

24. Approximate number of prenatal patients seen in a prenatal setting per week: ________________

25. Approximate percentage of your patients who are 'high-risk' for Down syndrome: ______________

26. Do you have experience in prenatal diagnosis for Down syndrome? ☐ Yes ☐ No

27. Do you currently offer NIPT? ☐ Yes ☐ No

27.1. If yes – to whom? (check all that apply)

☐ All pregnant women

☐ Women with pregnancies at high risk for Down syndrome after screening

☐ Women with pregnancies at high risk for aneuploidies based on ultrasound findings

☐ Other: ____

28. What type of Down syndrome screening do you currently offer to your patients?
(PLEASE CHECK ALL THAT APPLY)

☐ First trimester screening
(NT, free β-hCG, PAPP-A, MA)

☐ Quad screening
(AFP, uE3, free β-hCG, inhibin A, MA)

☐ Integrated prenatal screening (IPS)
(NT, PAPP-A, AFP, uE3, free β-hCG/total hCG, inhibin A, MA)

☐ Serum IPS
(PAPP-A, AFP, uE3, free β-hCG/total hCG, inhibin A, MA)

☐ Triple screening
(AFP, uE3, total hCG, MA)

☐ NIPT

☐ Other: ___________________________

Thank you for completing this survey.

If you have any additional comments or thoughts, please write them below.

© Vardit Ravitsky and Anne-Marie Laberge

INFORMATIONAL SHEET

Down syndrome (DS) is a genetic condition caused by the presence of an extra chromosome 21 (also called 'trisomy 21') which affects 1 in 770 newborns. Individuals with DS usually share physical features that are characteristic of DS. All have some degree of intellectual disability, which varies from person to person; their development is slower than other kids, but they will eventually learn to walk, talk, and dress themselves. Most children attend their neighborhood schools, some in regular classes and others in special education classes. Some children have more significant needs and require a more specialized program. Many adults with DS are capable of working in the community, but some require a more structured environment. Many will also have other health problems (for example heart defects). 99% of cases of DS are not inherited from the parents; it usually occurs by chance.

There are ways to check during pregnancy if there is a possibility that the baby has DS:

	**MATERNAL SERUM SCREENING (MSS or 'current screening')**	**AMNIOCENTESIS**	**NIPT**
**Description of the procedure**	• Checks the **level of risk** for DS<br>• Measures the **level of hormones** produced by the baby or placenta that end up in the mother's blood<br>• Includes **one or two blood draws** from the mother<br>• Where available, an ultrasound is done early in the pregnancy to measure nuchal translucency (level of fluid at the nape of the baby's neck)	• Medical procedure that can **confirm DS** during the pregnancy<br>• Allows checking the **number and appropriate structure of all chromosomes** in the baby's cells<br>• Requires **inserting a thin needle into the uterus** – through the mother's abdomen - to extract amniotic fluid (fluid in which the baby floats in the mother's womb)	• Checks the **level of risk** for DS<br>• Analyses the **baby's DNA** that is floating in the mother's blood<br>• Includes **one blood draw** from the mother
**Timing: When in pregnancy**	• 1st blood draw: usually between the **10th and 13th week** of pregnancy<br>• 2nd blood draw: usually between the **15h and 16th** week of pregnancy<br>• Results can be available between the **16th and 17th** week of pregnancy	• Available from the **15th week** of pregnancy<br>• Results can be available between the **17th and 19th week** of pregnancy	• Available as of the **10th week** of pregnancy<br>• Results can be available between the **11th-12th week** of pregnancy
**Risk to pregnancy**	**No increased risk of miscarriage**	**Risk of miscarriage** around 1 in 200 (0.5%)	**No increased risk of miscarriage**
**Accuracy**	Detects between 77% and 88% of DS cases (supported by a lot of evidence)	100% accurate in detecting DS (supported by a lot of evidence)	98% accurate (or more) for DS in women who are considered "high risk" based on MSS (supported by some evidence)
**Type of test**	Screening	Diagnostic	Screening
**What it detects**	• Down syndrome<br>• Trisomy 18<br>• Neural tube defects (e.g. spina bifida)<br>• Possible pregnancy complications	• Down syndrome<br>• Trisomy 13<br>• Trisomy 18<br>• Other chromosome anomalies<br>• Neural tube defects (e.g. spina bifida)<br>• Sex of the baby	• Down syndrome<br>• Trisomy 13<br>• Trisomy 18<br>• Sex of the baby
**Possible outcome**	• The test can predict that the pregnancy is at:<br>→ **Low risk** for DS (less than 1/200 – 1/300) so no further test is recommended<br>→ **High risk** for DS (higher than 1/200 – 1/300)<br>• **Amniocentesis is** offered to check if the baby actually has DS or other abnormalities detectable by chromosome analysis.	• **Normal result**: the baby does not have DS and has normal chromosomes.<br>• **Abnormal result**: the baby has DS or has another significant chromosome abnormality. In this case, the parents can choose to:<br>→ **continue the pregnancy**<br>→ **stop the pregnancy**	• The test can predict that the pregnancy is at:<br>→ **Very low risk** for DS - so no further test is recommended<br>→ **Very high risk** for DS<br>• **Amniocentesis is recommended** to confirm that the baby has DS

Note: NIPT is not yet considered as a first-tier screening test (i.e. that could replace serum screening); the current available evidence supports its use as a second tier screening test – after a positive serum screening and before an amniocentesis.

Last update: July 15, 2014
© PEGASUS: Vardit Ravitsky, Anne-Marie Laberge
